# Long-term ultrasonographic changes of the canine prostate gland after castration

**DOI:** 10.3389/fvets.2024.1524896

**Published:** 2025-01-08

**Authors:** Stefano Spada, Daniela De Felice, Sebastian Arlt, Luiz Paulo Nogueira Aires, Gary C. W. England, Marco Russo

**Affiliations:** ^1^Department of Veterinary Medicine and Animal Production, University of Naples, Federico II, Naples, Italy; ^2^Clinic of Reproductive Medicine, Vetsuisse Faculty, University of Zurich, Zurich, Switzerland; ^3^School of Agricultural and Veterinarian Sciences, São Paulo State University “Júlio de Mesquita Filho” (FCAV/UNESP), São Paulo, Brazil; ^4^School of Veterinary Medicine and Science, University of Nottingham, Nottingham, United Kingdom

**Keywords:** castration, canine prostate, CEUS, B-mode ultrasound, involution

## Abstract

**Introduction:**

Ultrasound imaging (US) is the method of choice to assess the canine prostate gland. Whilst recent studies have documented the role of castration in the development of prostatic neoplasia, little is known about parenchymal and perfusion features of the normal and abnormal prostate in neutered dogs. No data are available concerning prostatic changes after the first 90 days following castration. The present study aimed to acquire data on the long-term ultrasonographic changes occurring to the canine prostate after castration.

**Materials and methods:**

Ten adult neutered dogs underwent B-mode US and contrast-enhanced ultrasound (CEUS) of the prostate on two occasions: day of the first examination (T0) and six years later (T1). The prostate was evaluated via B-mode US and the volume was calculated using Atalan’s formula. For CEUS examination, an intravenous contrast agent (SonoVue) was administered to assess prostatic perfusion. Videoclips were recorded, and time-intensity curves were obtained to determine contrast parameters: peak enhancement (PPI) and time to peak (TTP). Volumetric and perfusion results were then compared between timepoints.

**Results:**

At both examinations, the prostate appeared similar on B-mode US in terms of morphology and echotexture, minimally decreasing in volume over time. Prostate perfusion was significantly reduced in all dogs between T0 and T1, with a PPI decrease from 54.9 to 29.6% and an increase in TTP from 26.3 to 47 s.

**Discussion:**

These preliminary data provide baseline information on the B-mode appearance and CEUS measurements of the prostate gland of neutered dogs and suggest that prostatic involution after castration is not a short-term process but continues over several months.

## 1 Introduction

Contraception by surgical sterilization including castration is an irreversible procedure that results in permanent cessation of reproductive function. As castration is irreversible, such surgical programs are widely accepted for population control (1). Bilateral castration has a prophylactic and therapeutic effect on androgen-dependent diseases, such as benign prostatic hyperplasia (BPH), chronic and acute prostatitis and perineal hernias (1–3). In dogs, BPH may be diagnosed on 80% of intact male dogs over 5 years of age (4) and more than 95% of intact male dogs over 9 years old (3).

The prostate gland is an androgen-dependent organ and castration results in regression of prostatic tissue in both normal dogs and dogs with BPH (5, 6). After removal of the testes, androgen concentrations decline, leading to a rapid involution of the size of the gland, with an expected reduction of 80% within 90 days, thus affecting prostate features and function (6, 7). Interestingly, whilst castration is an accepted and well-recognized method for the prevention and treatment of BPH (3), recent studies reported a higher risk of prostatic neoplasia in neutered dogs, thus increasing concern for choosing castration for the treatment of canine BPH. Nevertheless, it appears that castration does not initiate tumor development but may increase the incidence or hasten the progression of prostatic neoplasia (8–11). However, a definitive reason has not been identified yet, thus being imperative improving the knowledge of prostate physiology after gonadal hormone withdrawal and the changes that occur in the prostatic parenchyma.

Unfortunately, data about the ultrasonographic changes that occur throughout the period of prostate gland involution after castration are scant (5–7, 12). B-mode US is particularly useful for the assessment of size, shape, margins, echogenicity, echotexture and position of the prostate gland, as well as for evaluating the draining reproductive lymph nodes (2, 13–15). Despite the excellent application of US for imaging the prostate gland, the detection of prostate neoplasia can be challenging due to the lack of specific B-mode ultrasonographic features both in intact and neutered dogs (11, 13, 16, 17). Prostatomegaly, inhomogeneous parenchyma and the presence of mineralization are common but nonspecific findings of prostatic neoplasia, since such signs may be found also in non-malignant conditions, highlighting the importance of establishing reference characteristics in neutered male dogs (17). Moreover, few studies have been conducted on the ultrasonographic appearance and features of the normal prostate gland in dogs following castration (18–20).

Innovative ultrasonographic techniques, such as contrast-enhanced ultrasound (CEUS), have been successfully employed to improve the visualization and diagnosis of pathologic conditions of the prostate (13, 19–23). CEUS involves the intravenous injection of gas-filled microbubbles which enhance the backscatter signal of ultrasound waves, resulting in the amplification of the signals coming from blood flow (24, 25). Prostate neoplasia, even in its early stages, usually exhibits increased blood flow due to neoangiogenesis. These features may be detected by using CEUS, including asymmetric rapid inflow, increased focal enhancement, and asymmetry of intraprostatic vessels which are beyond the resolution of conventional Color or Power Doppler (13, 22, 26).

Prostatic perfusion features in healthy neutered dogs have been described, whereas information about vascularization of prostatic neoplasia in this group is still under investigation (19). Despite the promising results obtained by performing CEUS for the characterization of prostatic perfusion in dogs, it is still not considered widely as a diagnostic tool since there few studies have been conducted in this species. Furthermore, vascularization regression analysis and description after castrationhas been poorly investigated. Angrimani and colleagues reported that castration induces morphological and vascular involution, in terms of Doppler US parameters and histological findings (5). Yoon et al. (12) analyzed prostate perfusion changes using both CEUS and computerized tomography (CT) confirming the vascular regression occurring after castration. Nevertheless, the focus of all the studies was to monitor regression of the prostate in the first 90 days after castration, and to our knowledge no information is available about changes occurring after this period of time.

Since neutered dogs appear to be more prone to developing prostate neoplasia, we hypothesized that parenchymal changes may continue to occur beyond the first 90 days following castration. B-mode ultrasound and CEUS could provide valuable insights into prostate physiology in this group. Understanding these mechanisms is crucial to determine whether specific parenchymal changes occurring after castration contribute to or promote neoplastic transformation. This knowledge could enhance the diagnostic efficiency of detecting canine prostate pathologies following castration. The aim of the present study was to use B-mode and CEUS to monitor long-term prostate involution in a group of dogs at least three months following castration.

## 2 Materials and methods

### 2.1 Animal selection

Adult, mix breed, male neutered dogs were selected from a private kennel in Caivano (Naples, Italy). Inclusion criteria for the present study were as follows: the medical history of the dog should not have recorded any disease related to the urogenital system; no dogs with suspicion of neoplasia based on clinical signs, blood analysis and andrological examination should be included; the recruited dogs had to be older than 15 months of age when selected and the prostatic gland should have appeared within normal limits in terms of size, shape, margins, echogenicity and echotexture, with no focal lesions, in both the ultrasonographic examinations. Prostate glands were considered normal when volume did not exceed 38 cm^3^ (27), and when characterized by an ellipsoid shape, with smooth margins, hypoechoic and homogeneous echotexture; the recruited dogs should have been neutered at least 3 months prior the first examination (T0) performed within this study.

All dogs were housed in the kennel since the time of the castration, which was performed by public veterinary officers, as part of the public control of stray dogs. The reproductive history prior the castration was unknown.

### 2.2 Experimental design

The dogs were evaluated by using B-mode US and CEUS twice: the first evaluation was performed at the moment of the selection (T0) and the second one was performed approximately six years later (T1). At T0, sixty-four specimens were evaluated, and the results have been published in a recent study (19). Six years after T0, the remaining survived dogs were examined again (T1).

No sedation was required for examination as all dogs were cooperative. A standardized examination was performed for all dogs consisting of a recent and historical anamnesis of the patient, a general clinical examination followed by an andrological clinical examination, comprehensive of inspection and palpation of the prepuce, penis and scrotal remnants if present, digital rectal palpation and B-mode US and CEUS of the prostate gland. The animal study was approved by Ethical Animal Care and Use committee, University of Naples Federico II, Department of Veterinary Medicine and Animal Production (protocol 2016/0090751). The study was conducted in accordance with the local legislation and institutional requirements.

### 2.3 B-mode ultrasound

For the US examination, dogs were positioned in right lateral recumbency, to the right of the operator and with the head parallel to the US machine. Prepubic hairs were clipped, and alcohol and acoustic gel were applied to the skin. B mode and CEUS evaluations were performed with two different sets of equipment: a microconvex transducer (2–8 MHz) for B-mode US and a high frequency linear transducer (4–9 MHz—Esaote Mylab 30 gold, Genova, Italy) for CEUS at T0; a multifrequency microconvex transducer (3–11 MHz—Mindray, mQuadro, Vetus 7, Mindray Medical, Milan, Italy) for both B-mode and CEUS at T1. The ultrasonographic procedure was performed by a single operator at both timepoints (MR).

Image quality adjustments (gain, depth, focal zone, dynamic range) were made as necessary to obtain optimal quality images. For B-mode US, the microconvex transducer was placed on the skin and the urinary bladder was visualized. Then, the probe was moved caudally until a detailed image of a longitudinal bilobed prostate gland was acquired.

For the size of the prostate gland the following linear measurements were acquired:

- In the longitudinal view:
  - Length (L – Cranio-caudal length);
  - Depth (DL – Dorso-ventral length).
- In transverse view:
  - Width (W – Latero-lateral length);
  - Transverse depth (DT – Dorso-ventral length).

In order to acquire a correct transverse view of the gland, the probe was rotated of 90°. For all the measurements the maximum length was always acquired being careful to respect orthogonal plane.

Prostate volume (PV) was then calculated by using the Atalan formula (28):

$$PV=\frac{0.487\times L\times W\times \left(DL+DT\right)}{2}+6.38$$

The prostate was evaluated in terms of size, shape, margins, echogenicity, and echotexture in both views. Colour Doppler was then activated, and sample region was placed on the prostate gland. PRF (pulse repetition frequency), WF (wall filter) and gain were adjusted to improve the quality of the examination.

### 2.4 CEUS examination

CEUS examination procedure was similar to the one described in the previous studies conducted on the examination of perfusion kinetics in canine prostate gland (13, 19–22). For CEUS examination, a 20G intravenous, three-way valved catheter (Smiths Medical Jelco, Lower Pemberton, Ashford, Kant, UK) was placed in the cephalic vein to allow rapid infusion of the bolus dose of a freshly prepared second-generation contrast agent SonoVue^®^ (sulfurhexafluoride microbubbles; Bracco Imaging S.p.A., Milan, Italy). Both the transducer used for CEUS examination were characterized by coded harmonic capability and equipped with a dedicated software for CEUS analysis (Contrast Tuned Imaging (CnTI-TM), Contrast Tuned Imaging Technology, Esaote, Genova, Italy at T0; UWN+ (Ultra-Wideband Non-linear Contrast Imaging Feature) Contrast ImagingTM at T1). For each examination the mechanical index was set lower than 0.1 (range 0.05–0.1), corresponding to an acoustic pressure of 45 kPa, to decrease the acoustic impact of the ultrasound waves on the microbubble contrast agent. The prostate gland was imaged, and a single focal zone was placed covering the whole prostate. Dual visualization at T1 allowed the creation of a reference B-mode image on the screen once the contrast software was activated. At T0, gain and time-gain compensation were adjusted to result in a reduced signal from the parenchyma. The contrast agent, prepared according to the manufacturer’s manual, was injected into the cephalic vein at a dose of 0.03 mL/kg of solution (5 mg/mL), directly followed by a rapid bolus of 5 mL of saline solution. The timer was activated at the moment of the beginning injection (t = 0), and the flow of the contrast agent into the prostate was observed in real time. Care was taken to keep the probe in the same position for at least 60 s. The entire examination was digitally recorded, to be reviewed, so that the enhancement pattern could be systematically analyzed. The examinations were reviewed and evaluated subjectively by an experienced operator, which compared the examination between T0 and T1, blinded from the quantitative analysis of the video.

For the advanced quantitative evaluation of the CEUS data, two commercial software programmes, Qontrast^®^ (EC mark nr.0051, class IIA, Bracco, Milan, Italy) and Mindray machine software (UWN + Contrast ImagingTM Quantification Analysis Software) were used, respectively, at T0 and T1, to design time–intensity curves. In each frame, the entire prostate was used as a single region of interest (ROI), manually defined by drawing a line around the shape of the prostate. The software calculated time–intensity curves on a pixel-by-pixel basis, fitted them to parametric curves, and calculated the following parameters, starting from the moment of injection: perfusion peak intensity (PPI) expressed as a percentage, and Time to Peak (TTP, starting from t = 0) expressed in seconds. Median values and percentile quartiles of the perfusion parameters were then calculated.

The recruited dogs were monitored and observed for at least two hours after the procedure by the clinician in charge of the kennels, and during the following 24 h by the kennel staff to detect any immediate or delayed reaction to the contrast agent injection (29).

### 2.5 Statistical analysis

Data were recorded on a spreadsheet (Microsoft^®^ Excel^®^ 2021, Redmond, WA, United States) and then imported into Statistical Package for Social Sciences (SPSS IBM^®^ Statistics version 27.0, IBM Corporation, Armonk, NY, United States) for statistical analysis. Non-parametric tests were used for evaluation. *Post hoc* analysis with Wilcoxon’s signed-rank test was conducted to compare individual changes in terms of prostate volume, PPI and TTP between the two timepoints. Percentage of decrease or increase of the vascular parameters detected with CEUS was then calculated. The PPI and TTP percentage values, and regression rate of these parameters were then correlated to weight, age, age at castration and time elapsed since castration using Spearmen test, in order to detect any influence of individual parameters such as age, weight, age at castration and time elapsed since castration on the vascular changes occurring to the prostatic parenchyma such as PPI (T0), PPI (T1), TTP (T0), TTP (T1), PPI percentage change and TTP percentage change. Differences were considered statistically significant when *p* < 0.05.

## 3 Results

### 3.1 B-mode ultrasound

**Figure 1:**
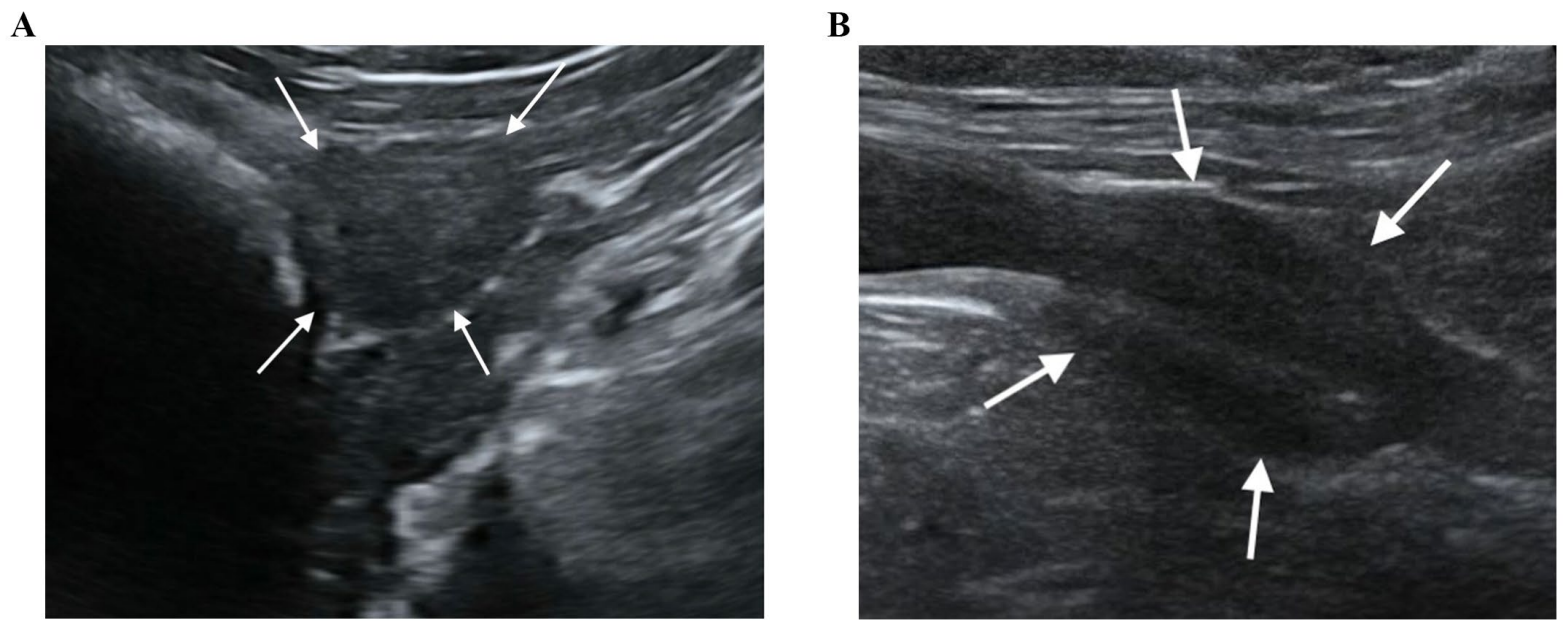
B-mode US of the prostate gland in transverse **(A)** and longitudinal view **(B)**. The prostate gland appears as a hypoechoic spheroidal **(A)** or ellipsoid **(B)** structure, with smooth margins and homogeneous parenchyma.

**Figure 2:**
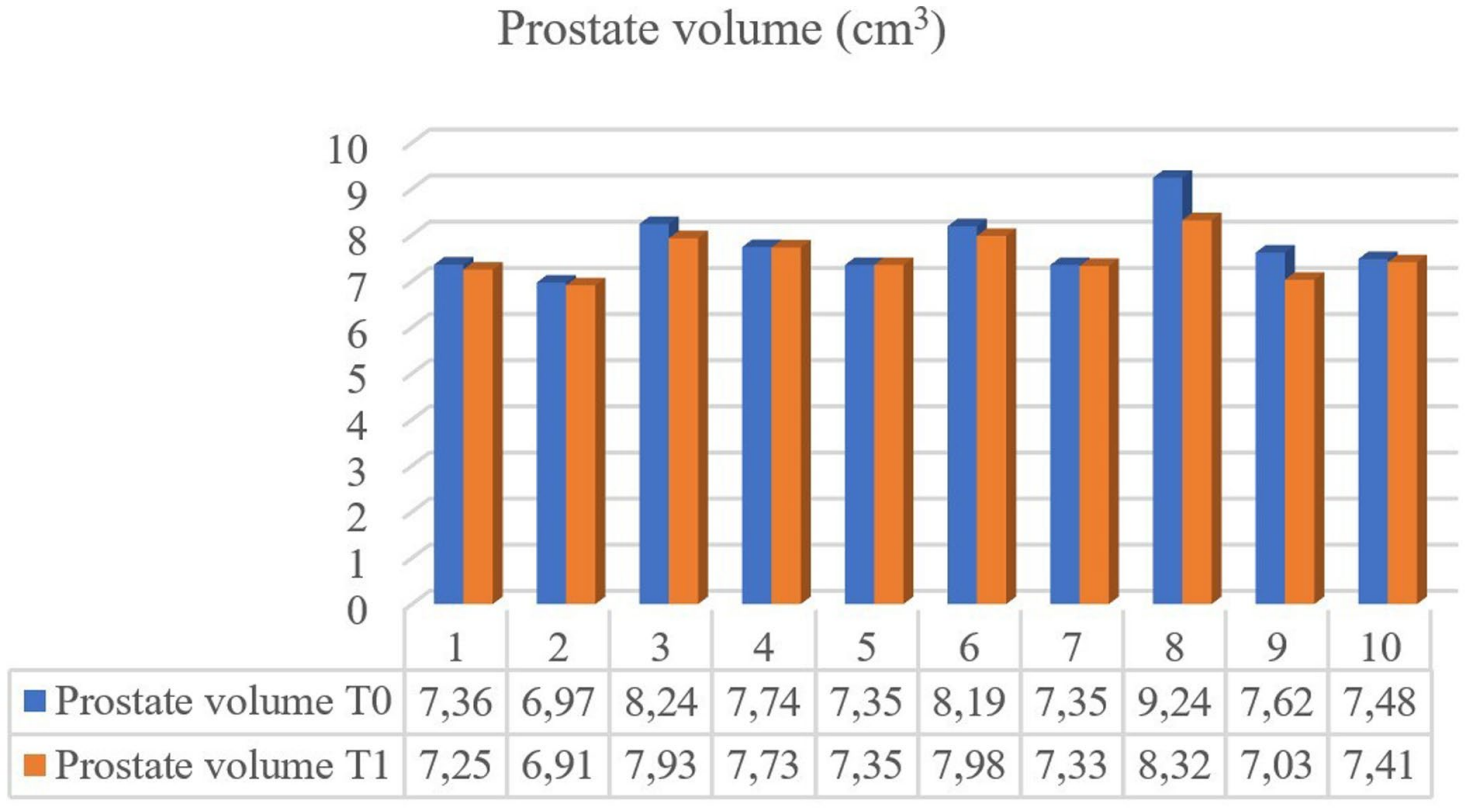
Prostate volume comparison for 10 individual dogs imaged after at least 3 months elapsed since castration (T0) and six years later (T1).

Of the 64 dogs evaluated at T0, just eleven were still alive at T1. However, ten healthy, neutered male, mix breed, kennelled dogs out of the eleven met the inclusion criteria and descriptive features of the sample population selected for this study are reported in Table 1. One dog had to be excluded from the present study for prostatic abnormalities detected at T1 correlated to a splenic mass with multiple metastasis including prostate gland. No physical or ultrasonographic abnormalities and no side-effects or anaphylactic reactions related to the CEUS examination were found in any of the ten patients. All prostate glands appeared as an ellipsoid shaped gland, with smooth margins and a hypoechoic homogenous parenchyma. The urethra was visible as a linear or circular hypoechoic structure on longitudinal or transverse view, respectively, in all dogs (Figure 1). No visible changes in the prostatic parenchyma were found in terms of echogenicity and echotexture between T0 and T1. A minimal decrease in prostate volume was detected (*p* = 0.005), with a median prostate volume at T1 of 7.39 cm^3^ (Table 2) and an average reduction in volume of 0.23 cm^3^ (Figure 2). Colour doppler US did not allow the detection of prostatic vascular flow in any of the patients at T0 and T1, even by using a low PRF (pulse repetition frequency) and higher Gain, it was not possible to visualize any vessel.

**Table 1:** Descriptive features of the sample population.

Dog number	Weight (kg)		Age (months)		Age at castration (months)	Time elapsed since castration (months)	
	T0	T1	T0	T1		T0	T1
1	15	17	24	96	11	13	85
2	10	10	36	108	13	23	95
3	24	22	60	132	44	16	88
4	23	25	36	108	11	25	97
5	24	24	36	108	14	22	94
6	24	23	48	120	26	22	94
7	38	42	48	120	25	23	95
8	32	29	63	135	60	3	75
9	9	9	72	144	51	21	93
10	9	9	36	108	12	24	96
Median (IQR)	23.5 (9.75–26.0)	22.5 (9.75–26)	42 (36–60.75)	114 (108–132.75)	19.5 (11.75–45.75)	22 (15.25–23.25)	94 (87.25–95.25)

**Table 2:** Median and interquartile ranges of prostate volume, PPI and TTP in T0 and T1.

	T0	T1
Prostate volume cm^3^	7.55 (7.35–8.2)	7.39 (7.19–7.95)_a_
PPI (%)	54.05 (45.8–64.07)	28.4 (18.85–35.66)_a_
TTP (s)	27.05 (21.7–32.05)	46.83 (38.41–53.69)_a_

Both prostate volume and PPI decreased, whereas TTP increased significantly from T0 to T1 (a = *p* < 0.01).

### 3.2 CEUS examination

**Figure 3:**
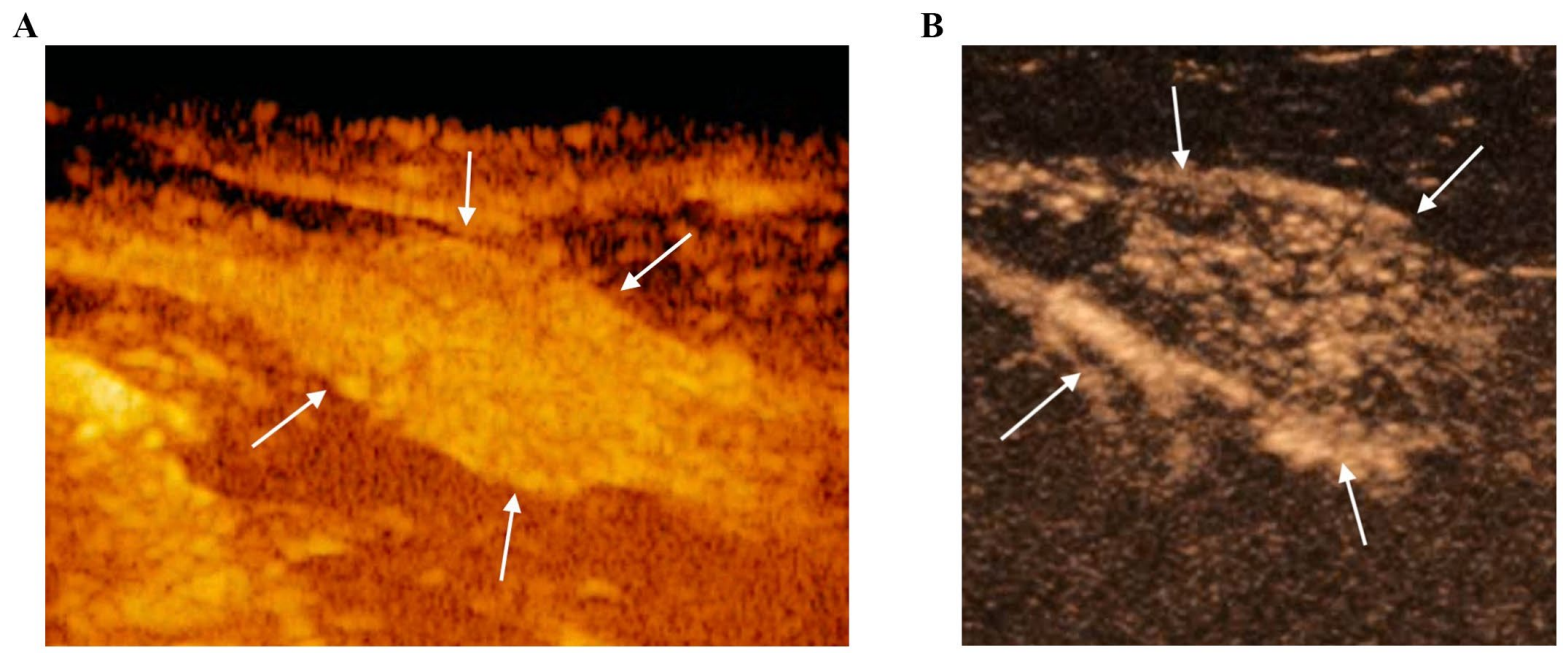
CEUS of the prostate gland (white arrows) at the peak enhancement performed at T0 **(A)** and T1 **(B)**. At T1 the prostate gland resulted to be less homogeneous and characterized by a reduced contrast intensity when compared to T0 by using CEUS.

**Figure 4:**
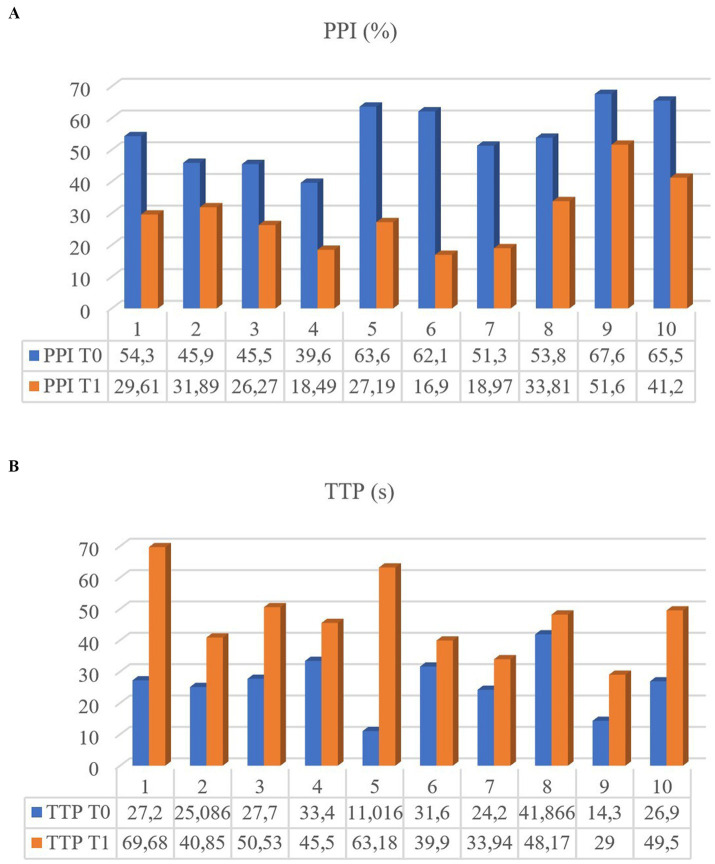
Perfusion Peak Intensity (PPI) **(A)** and Time to Peak (TTP) **(B)** of the 10 dogs measured at T0 and T1.

CEUS of the prostate did, however, reveal gland vascularization in all dogs. No differences were subjectively observed in the way the contrast agent enhanced the gland between the two different timepoints. The contrast agent firstly enhanced the signal of prostatic artery branches, which advance into the prostatic capsule and branched homogeneously into many small parenchymal arteries directed medially towards the urethra. In the wash-out phase, the parenchyma lost the enhancement pattern homogeneously. At T1 the prostate appeared subjectively less enhanced and brilliant when compared to T0 and contrast agent perfusion resulted to be slower and less homogeneous (Figure 3). Quantitative analysis confirmed and unveiled a significant reduction in PPI by an average of 46.26% (*p* = 0.005), alongside a notable increase in TTP by an average of 107.95% (*p* = 0.005) from T0 to T1 in all dogs. Contrast parameters comparisons are represented in the graphs (Figure 4). Correlation testing revealed a significant moderate positive correlation (Rho = 0.7) between weight and the percentage decrease in PPI (*p* = 0.028). No further correlations were identified between the percentage increase of TTP and individual parameters.

## 4 Discussion

The findings from the present study provide a baseline of information on the B-mode appearance and CEUS measurements of the prostate gland in dogs following castration. The mean time elapsed since castration at T0 was approximately 2 years, with a subsequent examination conducted 6 years later. Notably, the measured features varied between these two time points, indicating that prostatic involution is not a short-term process. These preliminary results suggest ongoing changes in the prostate for an extended period after castration. In particular, we observed regression of prostate vascularization and a trend for prostate volume to reduce between the two time points after castration. Although the duration of involution could not be precisely estimated due to the long interval between imaging sessions, it clearly persisted well beyond 3 months after castrationwhich in the period examined in other studies (5, 6, 12). To our knowledge this is the first study to analyze long-term prostatic involution using US in dogs.

Prostatic involution, characterized by a reduction in prostate gland size, involves progressive shrinkage of prostatic acini with a relative increase in fibromuscular tissue, resulting in fewer tubules with a single epithelial lining (30). These findings are in accordance with the observed higher collagen fiber density of periurethral and peripheral region of the prostate gland in neutered dogs (31).

Castration is considered to be the fastest treatment for benign prostate conditions in dogs with the greatest rate of involution expected within one week after surgery (3, 6). Although a significant reduction in prostate volume was detected in the present study, the average percentage of volume regression was only 2.8%, which represents a minimal amount in terms of gland size. Interestingly, one dog exhibited nearly no size reduction since the previous examination. The present results are coherent with the findings by Yoon et al. (12) and Cazzuli et al. (6), on the timing of regression of the prostate gland, where a remarkable regression in the first 7–15 days after castration was described, followed then by a decrease in the involution rate, with minimum percent volume loss recorded between 60 and 90 days.

We hypothesize that prostate size involution slows remarkably after the initial 3-month period following castration, until minimal percentage of volume reduction is reached. However, since our study involved examinations at only two time points with a six-year interval between T0 and T1, we cannot further delineate the regression during specific time intervals. In this study, we opted to focus solely on prostate volume rather than individual linear measurements of the gland, even though many variables are considered within the calculation (18, 28), due to the fact that prostatic enlargement has been observed to be non-uniform in dogs (32). The prostate volume results of 7.39 cm^3^ for neutered dogs are in line with findings from other studies (12, 19). Besides recent studies have reported that prostatomegaly may not be the sole indicator for diagnosing prostatic malignancies (17), establishing a baseline for mean dimensions in neutered dogs remains crucial for early detection of prostate changes.

Since parenchymal changes are often not sensitive enough for the detection of prostatic neoplasia, the assessment of vascular blood supply abnormalities of the gland has the potential to be helpful for an early diagnosis (13, 22). Prostate gland vascularization is significantly influenced by neuter status and hormonal activity. Several studies have investigated vascular parenchymal changes during benign and malignant conditions in intact dogs (5, 22, 23, 33, 34) and after castration (5, 12, 19).

CEUS has been successfully applied to the prostate gland in neutered dogs to describe prostate enhancement and perfusion, reporting differences between intact and neutered individuals. The present study documented similar perfusion characteristics detected in the previous studies conducted on prostate gland in neutered dogs (12, 19, 20). Notably, CEUS was able to detect vascularization in the present groups unlike Color Doppler. However, it remains uncertain whether this technique could serve as a standalone method or be combined with biopsy for early detection of prostate malignancies, as investigated in studies within human medicine (35–37).

To the best of our knowledge, this is the first study to report long-term regression of prostatic vascularization in dogs after castration, a phenomenon that extends beyond than expected. Lower PPI values are associated with a decreased vascular area and subsequently an overall reduction in blood perfusion. Conversely, an increase in TTP may be related to reduced number and diameter of the vessels branching off the prostate gland, leading to a slowdown in blood flow and, consequently, a delay in the enhancement of the prostate gland by the contrast agent.

In the present study, marked PPI decrease and TTP increase was observed in all dogs. These results support the findings from Yoon et al. (12), who noted a significant decline in vascular blood flow as early as 60 days post-castration using CEUS, a duration extending beyond the process of size involution. However, the difference in terms of regression rate between volume and vascular blood supply has not yet been clarified. Interestingly, vascular regression rates varied among individual dogs, with an average PPI decrease of 46.6% and a more than twofold increase in TTP compared to previous values. These results suggest that vascular regression following castration varies on an individual basis, that remains still unknown. Furthermore, a correlation was observed between weight and PPI regression rate, indicating a more pronounced vascular involution in larger dogs. These findings align with those of our previous study (20). Using different ultrasonographic machines between T0 and T1 may represent a potential limitation for the present study, especially when comparing results. However, even though numerical value may slightly change between the analysis of two different software, both CEUS examinations were firstly evaluated subjectively by an experienced operator. Quantitative analysis confirmed subjective observations concerning the reduced and slower enhancement observed in the examination in T1 compared to T0. Moreover, to ensure consistency in our results despite the use of different transducers, we adhered to a standardized protocol, utilized the same contrast agent, involved the same operator, and examined the same dogs. Additionally, parameters such as mechanical index, transducer frequency, and dynamic range were kept consistent across both evaluations. Castration results in an abrupt decrease in testosterone and dihydrotestosterone serum concentration, which are key regulators of prostate physiology and cell proliferation. Hence, decreased hormone concentration is followed rapidly by a significant prostate volume loss (10, 34). Notably, decreased androgen levels may also play a critical role in reducing blood perfusion, a major factor believed to influence cell proliferation post-castration (10). Recent studies reported that reduced dihydrotestosterone concentrations lead to lower Vascular Endothelial Growth Factor-A expression and thus definitely inhibiting neo-vascularization (5). Nevertheless, the difference in timing between morphological and vascularization involution may suggest that apoptotic processes occurring to vascular endothelial cells after castration may not be the main cause for cell proliferation arrest (12). Another reason could be that pre-acinar capillaries in the parenchymal zone may be the first vessels to involute, determining a severe reduction in terms of prostate epithelial cells nutrition and consequent apoptosis. The small size of the pre-acinar capillaries [with diameters of 5–7 μm and the endothelial layer thickness of 0.2 μm (38)] may explain why androgen deprivation effects on parenchymal perfusion enhancement may not be seen quickly after castration and may need more time before they are detected. It remains open, to which extent the changes documented in this study are caused by castration and the loss of sexual steroids. Potentially, the regression of prostatic volume and vascularization may also be partly dependent on age related changes. The absence of a control group of intact dogs represents a potential limitation in addressing this issue. However, since 90% of intact dogs over nine years of age develop BPH, we believe that the prostatic parenchymal changes observed in this group are significantly different from those occurring in neutered individuals, where hormone-linked conditions are absent. Nevertheless, further studies should investigate if similar changes may also be found in non-neutered male dogs.

## 5 Conclusion

The present preliminary study provides new insight into the B-mode appearance and CEUS measurements of the prostate gland after castration. The work provides a baseline of information for clinical practice that may be useful for differentiating clinical disease but also offers some novel perspective on prostatic physiology after castration.

## Data Availability

The original contributions presented in the study are included in the article/supplementary material, further inquiries can be directed to the corresponding author.

## References

[1] Reichler I. Gonadectomy in cats and dogs: a review of risks and benefits. Reprod Domest Anim. (2009) 44:29–35. doi: 10.1111/j.1439-0531.2009.01437.x, PMID: 19754532

[2] Smith J. Canine prostatic disease: a review of anatomy, pathology, diagnosis, and treatment. Theriogenology. (2008) 70:375–83. doi: 10.1016/j.theriogenology.2008.04.039, PMID: 18514299

[3] Cunto M Ballotta G Zambelli D. Benign prostatic hyperplasia in the dog. Anim Reprod Sci. (2022) 247:107096. doi: 10.1016/j.anireprosci.2022.107096, PMID: 36279818

[4] Sirinarumitr K Johnston SD Kustritz MVR Johnston GR Sarkar DK Memon MA. Effects of finasteride on size of the prostate gland and semen quality in dogs with benign prostatic hypertrophy. J Am Vet Med Assoc. (2001) 218:1275–80. doi: 10.2460/javma.2001.218.1275, PMID: 11330612

[5] Angrimani DSR Francischini MCP Brito MM Vannucchi CI. Prostatic hyperplasia: vascularization, hemodynamic and hormonal analysis of dogs treated with finasteride or orchiectomy. PLoS One. (2020) 15:e0234714. doi: 10.1371/journal.pone.0234714, PMID: 32584842 PMC7316311

[6] Cazzuli G Damián JP Molina E Pessina P. Post-castration prostatic involution: a morphometric and endocrine study of healthy canines and those with benign prostatic hyperplasia. Reprod Domest Anim. (2022) 57:157–64. doi: 10.1111/rda.14036, PMID: 34724270

[7] Johnston SD Kamolpatana K Root-Kustritz MV Johnston GR. Prostatic disorders in the dog. Anim Reprod Sci. (2000) 60-61:405–15. doi: 10.1016/S0378-4320(00)00101-9, PMID: 10844211

[8] Teske E Naan EC van Dijk EM Van Garderen E Schalken JA. Canine prostate carcinoma: epidemiological evidence of an increased risk in castrated dogs. Mol Cell Endocrinol. (2002) 197:251–5. doi: 10.1016/S0303-7207(02)00261-7 12431819

[9] Bryan JN Keeler MR Henry CJ Bryan ME Hahn AW Caldwell CW. A population study of neutering status as a risk factor for canine prostate cancer. Prostate. (2007) 67:1174–81. doi: 10.1002/pros.20590, PMID: 17516571

[10] Shidaifat F Gharaibeh M Bani-Ismail Z. Effect of castration on extracellular matrix remodeling and angiogenesis of the prostate gland. Endocr J. (2007) 54:521–9. doi: 10.1507/endocrj.K07-009, PMID: 17527004

[11] Schrank M Romagnoli S. Prostatic neoplasia in the intact and castrated dog: how dangerous is castration? Animals. (2020) 10:85. doi: 10.3390/ani10010085, PMID: 31948021 PMC7022700

[12] Yoon S Alfajaro MM Cho K-O Choi U-S Je H Jung J. Perfusion change in benign prostatic hyperplasia before and after castration in a canine model: contrast enhanced ultrasonography and CT perfusion study. Theriogenology. (2020) 156:97–106. doi: 10.1016/j.theriogenology.2020.06.026, PMID: 32682181

[13] Russo M Vignoli M England GCW. B-mode and contrast-enhanced ultrasonographic findings in canine prostatic disorders. Reprod Domest Anim. (2012) 47:238–42. doi: 10.1111/rda.12059, PMID: 23279509

[14] Mayer MN Lawson JA Silver TI. Sonographic characteristics of presumptively normal canine medial iliac and superficial inguinal lymph nodes. Vet Radiol Ultrasound. (2010) 51:638–41. doi: 10.1111/j.1740-8261.2010.01710.x, PMID: 21158237

[15] Citi S Oranges M Arrighi E Meucci V Della Santa D Tommaso M. Sonographic evaluation of medial iliac lymph nodes-to-aorta ratio in dogs. Vet Sci. (2020) 7:22. doi: 10.3390/vetsci7010022, PMID: 32054128 PMC7158673

[16] Mattoon JS Nyland TG. Prostate and testes In: Mattoon JS Nyland TG, editors. Small animal diagnostic ultrasound. St. Louis, MO, USA: Saunders (2015). 608–33.

[17] Bradbury CA Westropp JL Pollard RE. Relationship between prostatomegaly, prostatic mineralization, and cytologic diagnosis. Vet Radiol Ultrasound. (2009) 50:167–71. doi: 10.1111/j.1740-8261.2009.01510.x, PMID: 19400462

[18] Bosma F Wijsman S Huygens S Passon-Vastenburg M. Ultrasonographic measurements of the prostate gland in castrated adult dogs. Acta Vet Scand. (2022) 64:15. doi: 10.1186/s13028-022-00634-1, PMID: 35804438 PMC9264550

[19] Spada S England GCW Vignoli M Carluccio A Russo M. Contrast-enhanced ultrasound imaging of prostate gland in neutered dogs. Animals. (2021) 11:559. doi: 10.3390/ani11020559, PMID: 33672723 PMC7924405

[20] Spada S Arlt S De Felice D England GCW Russo M. Digital postprocessing analysis of prostatic perfusion in neutered dogs. Vet Radiol Ultrasound. (2024) 65:208–18. doi: 10.1111/vru.13343, PMID: 38363188

[21] Russo M Vignoli M Catone G Rossi F Attanasi G England GCW. Prostatic perfusion in the dog using contrast-enhanced doppler ultrasound. Reprod Domest Anim. (2009) 44:334–5. doi: 10.1111/j.1439-0531.2009.01442.x, PMID: 19754598

[22] Vignoli M Russo M Catone G Rossi F Attanasi G Terragni R. Assessment of vascular perfusion kinetics using contrast-enhanced ultrasound for the diagnosis of prostatic disease in dogs. Reprod Domest Anim. (2011) 46:209–13. doi: 10.1111/j.1439-0531.2010.01629.x, PMID: 20546182

[23] Troisi A Orlandi R Bargellini P Menchetti L Borges P Zelli R. Contrast-enhanced ultrasonographic characteristics of the diseased canine prostate gland. Theriogenology. (2015) 84:1423–30. doi: 10.1016/j.theriogenology.2015.07.029, PMID: 26277703

[24] Dietrich CF Averkiou M Nielsen MB Barr RG Burns PN Calliada F. How to perform contrast-enhanced ultrasound (CEUS). Ultrasound Int Open. (2018) 4:E2–E15. doi: 10.1055/s-0043-123931, PMID: 29423461 PMC5802984

[25] Nogueira Aires LP Gasser B Silva P Del’Aguila-Silva P Yamada DI Carneiro RK. Ovarian contrast-enhanced ultrasonography and Doppler fluxometry in bitches during the postovulatory estrus and corpora lutea formation. Theriogenology. (2022) 194:162–70. doi: 10.1016/j.theriogenology.2022.10.009, PMID: 36265337

[26] de Souza MB Da SLDM Moxon R Russo M England GCW. Ultrasonography of the prostate gland and testes in dogs. Practice. (2017) 39:21–32. doi: 10.1136/inp.i6054

[27] Laurusevičius T Šiugždaitė J Juodžiukynienė N Kerzienė S Anskienė L Jackutė V. Comparative evaluation of diagnostic methods for subclinical benign prostatic hyperplasia in intact breeding male dogs. Animals. (2024) 14:1204. doi: 10.3390/ani14081204, PMID: 38672352 PMC11047341

[28] Atalan G Holt PE Barr FJ. Ultrasonographic estimation of prostate size in normal dogs and relationship to bodyweight and age. J Small Anim Pract. (1999) 40:119–22. doi: 10.1111/j.1748-5827.1999.tb03052.x, PMID: 10200922

[29] Seiler GS Brown JC Reetz JA Taeymans O Bucknoff M Rossi F. Safety of contrast-enhanced ultrasonography in dogs and cats: 488 cases (2002–2011). J Am Vet Med Assoc. (2013) 242:1255–9. doi: 10.2460/javma.242.9.1255, PMID: 23600783

[30] Palmieri C Fonseca-Alves CE Laufer-Amorim R. A review on canine and feline prostate pathology. Front Vet Sci. (2022) 9:881232. doi: 10.3389/fvets.2022.881232, PMID: 35720846 PMC9201985

[31] Ruetten H Wegner KA Romero MF Wood MW Marker PC Strand D. Prostatic collagen architecture in neutered and intact canines. Prostate. (2018) 78:839–48. doi: 10.1002/pros.23641, PMID: 29740846 PMC6356104

[32] Haverkamp K Harder LK Kuhnt NSM Lüpke M Nolte I Wefstaedt P. Validation of canine prostate volumetric measurements in computed tomography determined by the slice addition technique using the Amira program. BMC Vet Res. (2019) 15:49. doi: 10.1186/s12917-019-1778-z, PMID: 30717756 PMC6360749

[33] Zelli R Orlandi R Troisi A Cardinali L Polisca A. Power and pulsed Doppler evaluation of prostatic artery blood flow in Normal and benign prostatic hyperplasia-affected dogs. Reprod Domest Anim. (2013) 48:768–73. doi: 10.1111/rda.12159, PMID: 23505997

[34] Lima CB Angrimani DSR Flores RB Vannucchi CI. Endocrine, prostatic vascular, and proapoptotic changes in dogs with benign prostatic hyperplasia treated medically or surgically. Domest Anim Endocrinol. (2021) 75:106601. doi: 10.1016/j.domaniend.2020.106601, PMID: 33333452

[35] Liu G Wu S Huang L. Contrast-enhanced ultrasound evaluation of the prostate before transrectal ultrasound-guided biopsy can improve diagnostic sensitivity. Medicine. (2020) 99:e19946. doi: 10.1097/MD.0000000000019946, PMID: 32384441 PMC7220038

[36] Postema AW Scheltema MJV Mannaerts CK Van Sloun RJG Idzenga T Mischi M. The prostate cancer detection rates of CEUS-targeted versus MRI-targeted versus systematic TRUS-guided biopsies in biopsy-naïve men: a prospective, comparative clinical trial using the same patients. BMC Urol. (2017) 17:27. doi: 10.1186/s12894-017-0213-7, PMID: 28381220 PMC5382402

[37] Salib A Halpern E Eisenbrey J Chandrasekar T Chung PH Forsberg F. The evolving role of contrast-enhanced ultrasound in urology: a review. World J Urol. (2022) 41:673–8. doi: 10.1007/s00345-022-04088-y, PMID: 35969244

[38] Sun F Báez-Díaz C Sánchez-Margallo FM. Canine prostate models in preclinical studies of minimally invasive interventions: part I, canine prostate anatomy and prostate cancer models. Transl Androl Urol. (2017) 6:538–46. doi: 10.21037/tau.2017.03.61, PMID: 28725597 PMC5503961

